# Targeting mtor-dependent tumours with specific inhibitors: a model for personalized medicine based on molecular diagnoses

**DOI:** 10.3747/co.v16i1.406

**Published:** 2009-01

**Authors:** L. Furic, M. Livingstone, R.J.O. Dowling, N. Sonenberg

**Keywords:** Cell signalling, mtor, rapamycin, Lkb1, Fkbp12, tumour growth

## Abstract

Cancer cells are characterized by aberrant growth arising from deregulated signalling pathways. The mammalian target of rapamycin (mtor) pathway integrates multiple growth signals coming from both intracellular and extracellular cues. In this short review, we summarize what is known about the efficacy of targeting the mtor pathway to treat cancer patients, and we explain the rationale behind promising new inhibitors that could show more potent tumour growth inhibition than did the first generation of these drugs.

## 1. INTRODUCTION

Signal transduction pathways govern appropriately regulated cell growth, division, and survival in human tissues, but these pathways are often deregulated in aggressive cancers that show seemingly uncontrolled growth. This observation has led to the notion that if the molecular cause of a cancer can be determined, then the aberration on which a particular cancer depends could be directly targeted^1^.

The mammalian target of rapamycin (mtor) signalling pathway has proved to be an excellent model system for exploring this notion of personalized medicine, because numerous mutations resulting in hyperactivation of the pathway have been identified, the hyperactivation is itself demonstrated by multiple downstream biomarkers, and a specific potent mtor inhibitor, rapamycin, has a long been safely used to treat humans^2^. In this review, we discuss the mutations and molecular diagnosis of tumours with hyperactivated mtor signalling, clinical trials of rapamycin analogues, the rationale behind second-generation non-rapamycin-analogue mtor inhibitors, and the ongoing early-phase clinical trials using these agents.

## 2. ABERRANT ACTIVATION OF MTOR

**FIGURE 1:**
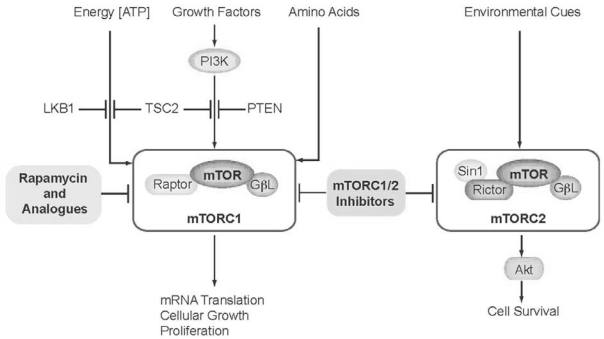
The phosphoinositide 3 kinase (PI3K)–Akt–mammalian target of rapamycin (mtor) pathway integrates multiple signals emanating from inside and outside the cell. The rapamycin-sensitive mtor complex 1 (mtorc1) responds to energy balance, growth factors, and availability of metabolites. The rapamycin-insensitive mtor complex 2 (mtorc2) promotes cell survival, but its regulation is less well characterized. atp = adenosine triphosphate; Lkb1 = serine/threonine kinase 11 (Stk11); Tsc2 = tuberous sclerosis 2 protein; pten = protein encoded by the phosphatase and tensin homolog gene; mrna = messenger rna.

The evolutionarily conserved (yeast to man) mtor protein kinase integrates multiple intracellular and extracellular signals representing growth conditions (energy, growth factor, and amino-acid availability, for instance) to control downstream processes, including translation of messenger rna, cell growth, and proliferation (FIGURE 1). Numerous mutations of upstream regulators of mtor signalling can lead to hyperactivation, thus uncoupling mtor activity from the cues that normally modulate its activity, resulting in unconstrained cell growth^3^. For example, the phosphoinositide 3 kinase (PI3K) signalling pathway is normally activated when growth factors bind transmembrane receptors; it is inactivated by pten (the protein encoded by the phosphatase and tensin homolog gene) when growth factors are not abundant. Similarly, serine/threonine kinase 11 [Stk11 (Lkb1)] negatively regulates mtor activity when cellular energy (adenosine triphosphate) is in low supply, and the tuberous sclerosis complex (Tsc1/Tsc2) negatively regulates both of those signals to mtor. Consequently, cells lacking functional pten, Stk11 (Lkb1), or Tsc2 exhibit deregulated, constitutive signalling to mtor, resulting in multiple types of cancer^1^–^4^.

## 3. RAPAMYCIN ANALOGUES: CLINICAL TRIALS

Rapamycin, a natural product from soil bacteria, inhibits mtor by simultaneously binding to a protein called Fkbp12 and to the Fkbp12 rapamycin-binding (frb) domain of mtor. Because of its ability to inhibit mtor-dependent growth and proliferation of B and T cells, rapamycin has long been used as an immuno-suppressant (sirolimus) to prevent organ rejection, and because of its ability to inhibit the fungal orthologue of mtor, it can also be used as an antifungal agent.

In clinical trials, rapamycin and three rapamycin analogues—CCI-779 [temsirolimus (Torisel: Wyeth-Ayerst, Charlotte, NC, U.S.A.)], RAD001 [everolimus (Certican: Novartis Pharmaceuticals, St. Louis, MO, U.S.A.)], and AP23573 (deforolimus: Ariad Pharmaceuticals, Cambridge, MA, U.S.A., and Merck and Co., Whitehouse Station, NJ, U.S.A.)—have been assessed for their efficacy as anticancer agents^5^,^6^. These compounds have been tested alone and in combination with standard-of-care agents for a wide variety of human cancers.

Based on preclinical testing, mtor inhibitors were predicted to be effective in the treatment of tumours with genetic mutations that lead to overactivation of mtor signalling—for example, loss of pten, Stk11 (Lkb1), or Tsc2. Cells and tumours containing mutations or deletions of these tumour suppressors exhibit uncontrolled mtor signalling and an increase in phosphorylation of the downstream targets Eif4Ebp1 (eukaryotic translation initiation factor 4E binding protein 1) and S6 kinase^7^–^9^. Data from clinical trials seem to support this hypothesis, because endometrial tumours, which exhibit a high frequency of pten inactivation, have responded favourably to treatment with temsirolimus^10^,^11^. Renal cell carcinoma also exhibits some sensitivity to mtor inhibitors, likely because of the importance of mtor signalling in the expression of Hif1A (hypoxia inducible factor 1, alpha subunit), a key player in angiogenesis and the growth of renal tumours^11^,^12^. In fact, results from a recent phase iii clinical trial involving patients with metastatic renal cell carcinoma indicate that everolimus may be an effective option for patients suffering from renal cancer^12^. Furthermore, temsirolimus was approved for the treatment of patients with renal carcinoma in May 2007^5^. This evidence indicates that overactive mtor signalling (as a result of genetic mutation, inactivation, or loss of key tumour suppressors) may be used to identify patients who would benefit from treatment with rapamycin analogues. However, although rapamycin analogues have been effective in the treatment of some tumours that lack pten (such as those of the endometrium), other cancers have been refractory to the effects of mtor inhibitors despite pten loss or mutation^13^,^14^. In these and other cases, results from clinical trials have been disappointing to some who had high hopes for this rationale-based chemotherapy^15^.

## 4. SECOND-GENERATION MTOR INHIBITORS

All of the above-described mtor inhibitors undergoing clinical trials are rapamycin analogues, and they therefore inhibit only a portion of mtor signalling—the portion effected by the rapamycin-sensitive mtor complex 1 (mtorc1)^16^. It has been hypothesized that inhibition of both mtorc1 and the rapamycin-insensitive mtor complex 2 (mtorc2) would be a more effective means of treating such tumours^17^,^18^, because inhibition of mtorc1 with rapamycin actually leads to an upregulation of mtorc2-dependent survival signalling through the PI3K-Akt pathway by suppressing a negative feedback loop^19^. Consistent with this reasoning, it has been shown that dual inhibition of mtor and PI3K–Akt is an effective means to target pten-deficient tumours^20^. Developing mtor inhibitors that target the kinase domain has been proposed as an effective means to block the activities of both mtorc1 and 2 mtorc^17^,^18^, and a new wave of clinical trials has commenced using a second generation of mtorc1 and 2 inhibitors. The agent OSI-027 (OSI Pharmaceuticals, Melville, NY, U.S.A.), for example, is an mtorc1 and 2 inhibitor currently being evaluated (phase i) to determine the maximum tolerated dose in patients with lymphoma or solid tumours. Similarly, BEZ235 (Novartis Pharmaceuticals) is being evaluated (phase i and ii) to determine dosing and effectiveness in treating advanced solid tumours, particularly those in patients with Cowden syndrome (caused by pten loss). A third mtorc1 and 2 inhibitor, XL765 (Exelixis, San Francisco, CA, U.S.A.) is being tested (phase i) alone and in combination therapies.

## 5. SUMMARY

As a whole, exploiting the mtor signalling pathway and its aberrant activation has provided strong proof that the age of personalized medicine based on molecular diagnoses is well underway. Progress to this point indicates that further elucidation of the mechanisms involved in predicting sensitivity to rapamycin and analogues is possible, as is development of new, more potent mtor inhibitors for the treatment of cancer. It is important to keep in mind that the signalling pathways inhibited by rapamycin analogues and by mtorc1 and 2 inhibitors are likely crucial for the growth, proliferation, and survival of possibly all human cells, and therefore such compounds should be effective treatments against most tumours in the same way that traditional chemotherapies are. However, the notion of “oncogene addiction” suggests that, if a particular aberration is driving tumour survival, then it may be possible to selectively target the cancer while merely inducing growth arrest or cytostasis in healthy tissues.
